# Strain-Stabilized (π, π) Order at the Surface of Fe_1+*x*_Te

**DOI:** 10.1021/acs.nanolett.0c04821

**Published:** 2021-04-02

**Authors:** Chi Ming Yim, Soumendra Nath Panja, Christopher Trainer, Craig Topping, Christoph Heil, Alexandra S. Gibbs, Oxana V. Magdysyuk, Vladimir Tsurkan, Alois Loidl, Andreas W. Rost, Peter Wahl

**Affiliations:** † SUPA, School of Physics and Astronomy, University of St. Andrews, North Haugh, St. Andrews, Fife KY16 9SS, U.K.; ‡ Tsung Dao Lee Institute & School of Physics and Astronomy, Shanghai Jiao Tong University, Shanghai 200240, China; § Institute of Theoretical and Computational Physics, Graz University of Technology, NAWI Graz, 8010 Graz, Austria; ∥ ISIS Neutron and Muon Source, STFC Rutherford Appleton Laboratory, Didcot OX11 0QX, U.K.; ⊥ School of Chemistry, University of St. Andrews, North Haugh, St. Andrews, Fife KY16 9SA, U.K.; # Max Planck Institute for Solid State Research, Heisenbergstrasse 1, 70569 Stuttgart, Germany; ∇ Diamond Light Source Ltd., Harwell Science and Innovation Campus, Didcot OX11 0DE, U.K.; ○ Center for Electronic Correlations and Magnetism, University of Augsburg, D-86159 Augsburg, Germany; ◆ Institute of Applied Physics, Academiei 5, MD 2028, Chisinau, Republic of Moldova

**Keywords:** Uniaxial strain, iron telluride, low-temperature scanning tunneling microscopy, charge order

## Abstract

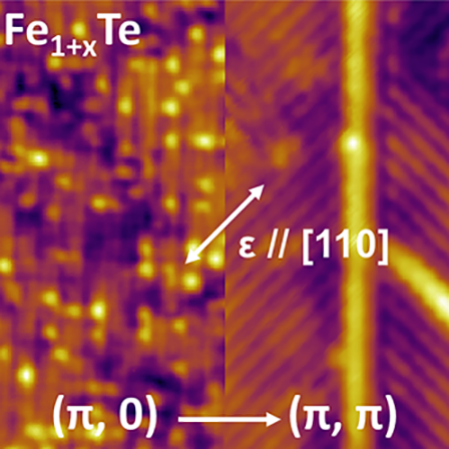

A key property of many quantum materials is that their ground state depends sensitively on small changes of an external tuning parameter, e.g., doping, magnetic field, or pressure, creating opportunities for potential technological applications. Here, we explore tuning of the ground state of the nonsuperconducting parent compound, Fe_1+*x*_Te, of the iron chalcogenides by uniaxial strain. Iron telluride exhibits a peculiar (π, 0) antiferromagnetic order unlike the (π, π) order observed in the Fe-pnictide superconductors. The (π, 0) order is accompanied by a significant monoclinic distortion. We explore tuning of the ground state by uniaxial strain combined with low-temperature scanning tunneling microscopy. We demonstrate that, indeed under strain, the surface of Fe_1.1_Te undergoes a transition to a (π, π)-charge-ordered state. Comparison with transport experiments on uniaxially strained samples shows that this is a surface phase, demonstrating the opportunities afforded by 2D correlated phases stabilized near surfaces and interfaces.

The interplay between magnetism and superconductivity is a common theme across iron-based superconductors.^1,2^ Most of them exhibit a (π, π)-ordered magnetic phase in some part of the phase diagram which is suppressed by chemical substitution until superconductivity sets in. This general behavior hints to the importance of magnetic fluctuations for superconductivity;^3^ yet, it is disrupted by the iron chalcogenides where the magnetic order occurs in the (π, 0) direction in Fe_1+*x*_Te^4−6^ and nematicity in the superconducting FeSe occurs without magnetic order,^7,8^ whereas in the pnictides nematicity and magnetic order are intimately linked and the magnetic order occurs at the same (π, π) scattering vector at which magnetic fluctuations dominate in the superconducting state. The difference in the magnetic order is also reflected in a different crystal structure in Fe_1+*x*_Te.^4−6^ Atomic scale imaging and spectroscopy provide a window into the local relation between magnetism, superconductivity, and the superconducting gap structure in iron-based superconductors.^9^ Here, we explore, using atomic scale imaging by scanning tunneling microscopy (STM) with uniaxial strain, whether the iron chalcogenides can be altered to behave in a similar fashion as the iron pnictides, by forcing the crystal lattice into an orthorhombic distortion through application of uniaxial strain.

Fe_1+*x*_Te cannot be grown in its stoichiometric form (with *x* = 0). At low interstitial Fe concentration (*x* ≤ 0.11), Fe_1+*x*_Te exhibits a bicollinear antiferromagnetic (AFM) order, characterized by a wave-vector of **q** = (π, 0, π) in reciprocal space.^4,5,10,11^ Varying the concentration of the interstitial Fe atoms within the sample leads to a variety of magnetic orders, accompanied by a distortion of the crystal lattice from monoclinic to orthorhombic with increasing *x*.^4−6,10,11^ Density functional theory (DFT) calculations have successfully predicted the commensurate bicollinear AFM structure at low *x*.^12,13^

Uniaxial strain tuning is a novel technique ideally suited to study symmetry-broken electronic phases in correlated electron systems.^14−16^ Unlike chemical doping that invariably introduces inhomogeneity, uniaxial strain alters the bulk electronic structure by small lattice distortion, in turn breaking their innate symmetry. Uniaxial strain can have a profound impact on their ground-state electronic properties. Notable examples include a strong increase in superconducting *T*_c_ of Sr_2_RuO_4_,^15^ a strain-induced 3D charge density wave-ordered state in YBa_2_Cu_3_O_6.67_,^17^ and the emergence of a charge-ordered electronic phase in LiFeAs.^16^

Here, we report a low-temperature STM study of the ground state of Fe_1.1_Te under small uniaxial strain. By applying strain along the [110] direction, we uncover a novel electronic phase, characterized by a wave-vector of (π, π) in reciprocal space. This newly observed (π, π)-ordered phase exhibits electronic and magnetic properties that are very different from the bicollinear AFM-ordered phase found in unstrained samples. We discuss their resemblance with the spin-density wave (SDW) ordered phase found in some of Fe-pnictides.

## Results

**Figure 1:**
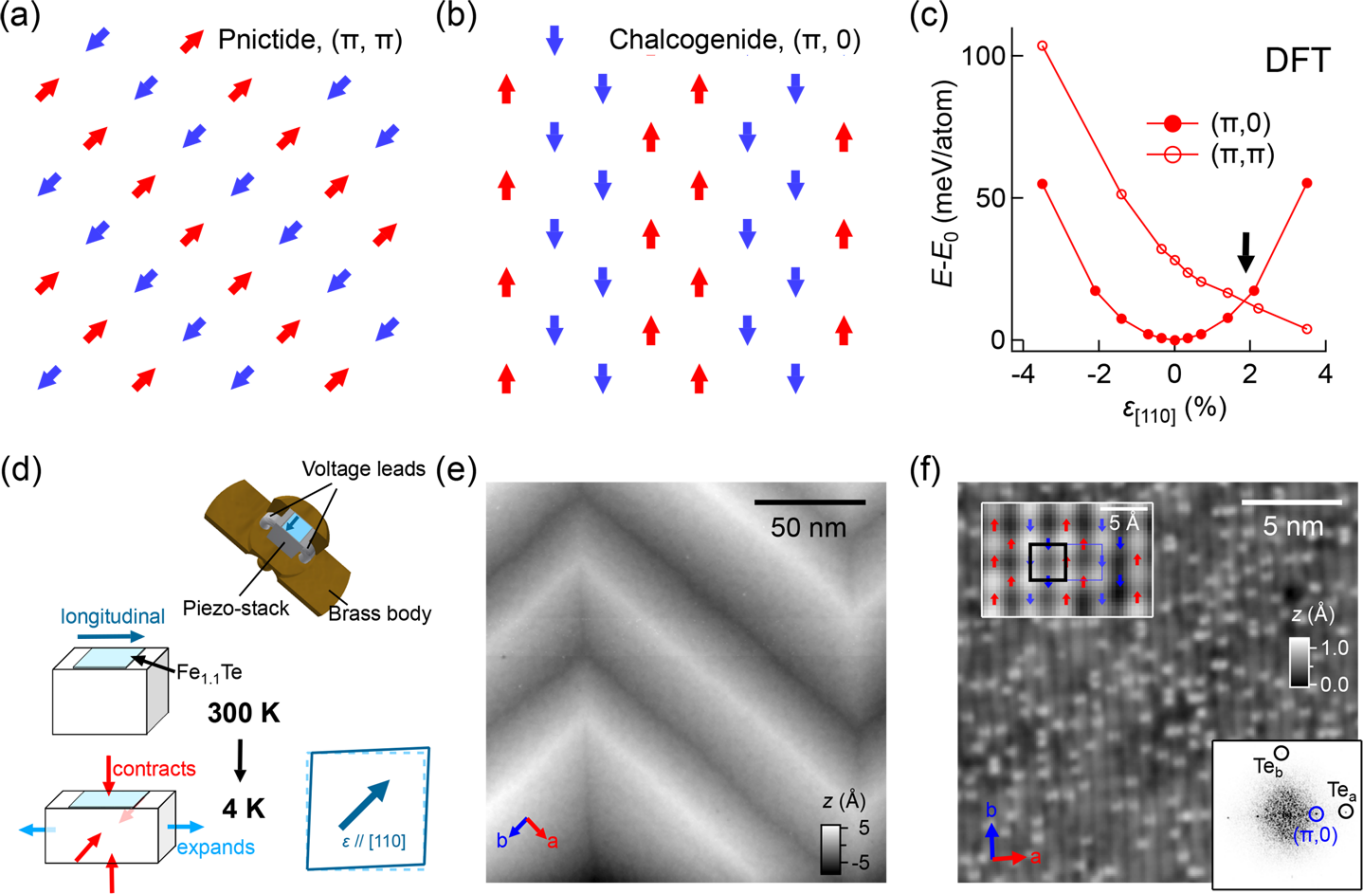
Surface magnetic orders in Fe-pnictides and chalcogenides. (a, b) Schematics of the (a) (π, π) and (b) (π, 0) bicollinear AFM orders present in Fe-pnictides and chalcogenides, respectively. Arrows represent the magnetic moments of the in-plane Fe atoms. (c) A scatter plot of the ground state energies of the two magnetic orders at different values of strain applied along the [110] direction, presented relative to the ground state energy of the (π, 0)-ordered phase at zero strain (defined as *E*_0_). Calculated within DFT, an arrow indicates the strain value beyond which the (π, π) order becomes more stable. (d) Schematic of the STM-strain setup, showing that due to the different thermal expansion properties of the sample and the piezo-stack, the sample is exposed to tensile strain along the longitudinal direction of the piezo-stack as the setup is cooled to *T* = 4 K. (e) STM image taken from the surface of an unstrained Fe_1.1_Te sample (*V* = 50 mV, *I* = 500 pA). (f) STM image taken within a monoclinic domain using a magnetic tip (*V* = 100 mV, *I* = 200 pA). Inset at bottom-right: Fourier transformation of (f). Inset at top-left: Image taken with a nonmagnetic tip (*V* = 200 mV, *I* = 50 pA), overlaid with arrows representing the (π, 0) spin-texture. A solid square (dashed rectangle) indicates the structural (magnetic) unit cell of Fe_1.1_Te.

The magnetic order in the iron pnictides (Figure 1a) and iron chalcogenides (Figure 1b) is correlated with a characteristic structural distortion. This suggests that distorting the crystal structure of Fe_1+*x*_Te toward that of the pnictides might change the magnetic order accordingly. This is confirmed by our DFT calculations performed on Fe_1+*x*_Te with no excess Fe interstitials (i.e., *x* = 0); under sufficient uniaxial strain along [110], FeTe adopts the same (π, π) magnetic order as seen in the iron pnictides. Figure 1c shows for comparison the energies of (π, π) and (π, 0) magnetic order in FeTe for different levels of strain along the [110] direction. At 2% uniaxial strain, the (π, π) order as found in the pnictides becomes more favorable. DFT tends to underestimate the influence of small lattice distortions on the electronic structure, so in reality the transition is expected to occur already for smaller levels of strain.

To test this idea, we have performed measurements at the atomic scale using low-temperature STM on Fe_1+*x*_Te samples under uniaxial strain applied along the [110] direction (see Experimental Section and Figure 1d for a schematic of the STM strain setup). The Fe_1+*x*_Te samples studied here are of low levels of excess Fe doping (*x* ≈ 0.1). As shown in the STM images in Figure 1, e and f, at these levels of doping, the samples adopt a monoclinic unit cell^10^ and exhibit a (π, 0) bicollinear AFM order.^10,11^

**Figure 2:**
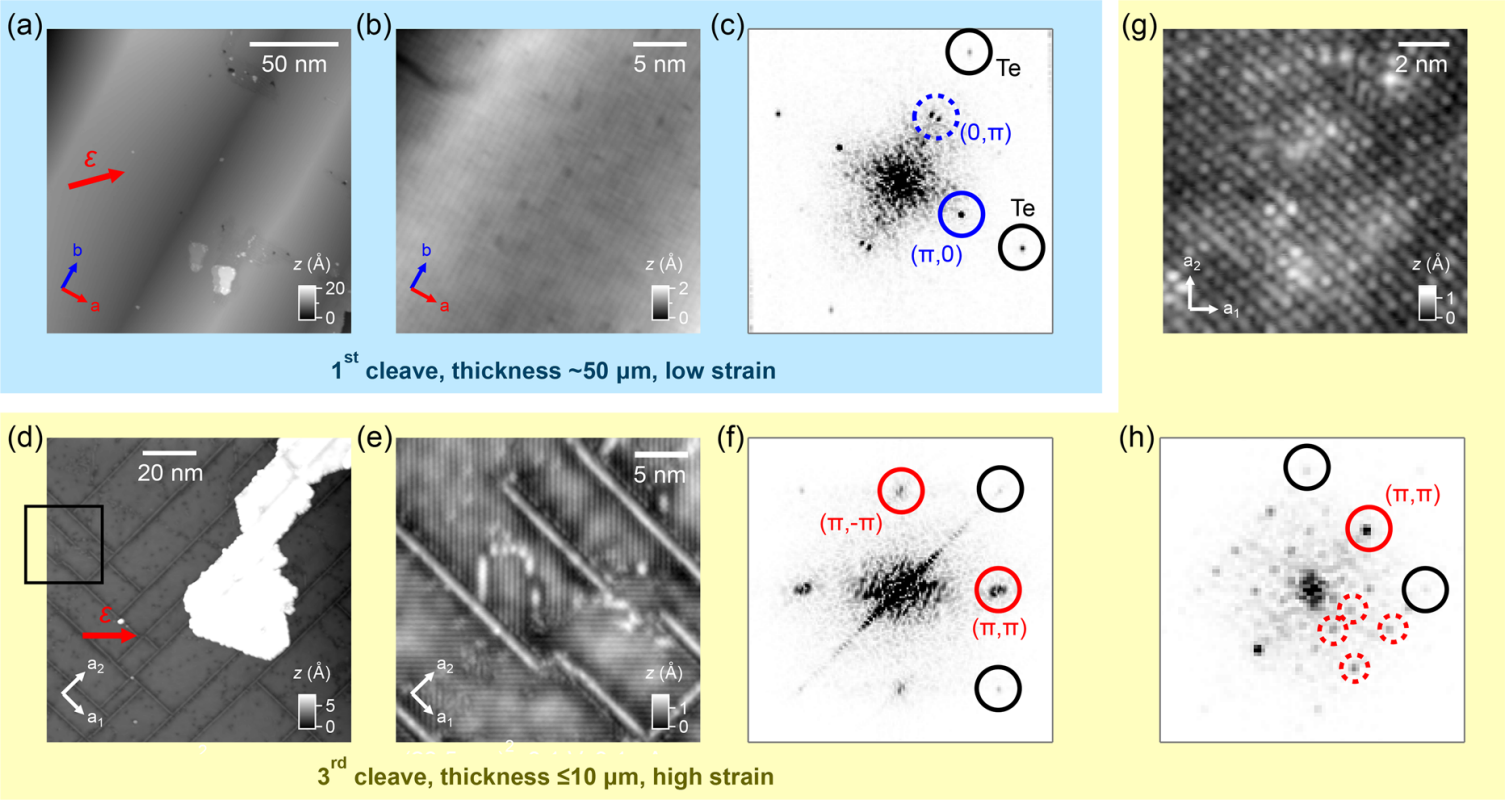
Surface morphology of a strained Fe_1.1_Te sample at decreasing sample thickness. (a) STM topographic image taken from the surface of a Fe_1.1_Te sample under uniaxial strain applied along the [110] direction (*V* = 200 mV, *I* = 200 pA). The sample has a starting thickness of 50 μm. The red arrow indicates the direction along which uniaxial strain is applied. (b) Atomically resolved image taken on the same surface as (a) (*V* = 20 mV, *I* = 200 pA), obtained using a magnetic tip. (c) Fourier transformation of (b) . Solid and dashed blue circles mark the (±π, 0) and (0, ±π) peaks that altogether form the checkerboard-like magnetic order. (d) Topographic image recorded after the third cleave (*V* = 200 mV, *I* = 100 pA, sample thickness ≈ 10 μm). (e) Zoomed-in image taken in a small region as marked with a square box in (d) (*V* = 100 mV, *I* = 100 pA). (f) Fourier transformation of (e) . (g) Atomically resolved image of the (π, π)-ordered phase (*V* = 10 mV, *I* = 100 pA) . (h) Fourier transformation of (g) . In (c), (f), and (h), black circles mark the Bragg peaks of the top-most Te lattice. Solid red circles mark the peaks of the (π, π) order. Dashed red circles mark the peaks of the superstructure(s) forming along the stripes.

Figure 2 shows topographic STM images taken at the surface of a Fe_1.1_Te sample mounted on a piezoelectric stack with increasing strain and decreasing sample thickness, starting from a thickness of ∼50 μm. Illustrated by the schematic of the strain setup (Figure 1d), due to the difference in thermal expansion of the sample and the piezoelectric stack, cooling the strain setup to the measurement temperature of 4 K leads to an upper limit of 0.3% of tensile strain present within the sample. Shown in Figure 2, a and b, the surface topography taken after the first cleave consists of monoclinic domains, which are separated from each other by domain boundaries. Different from the unstrained sample, the boundary lines here run along the crystallographic *b* direction only. Intriguingly, the surface no longer exhibits the (π, 0) bicollinear AFM order as it normally does in unstrained samples. The strained sample exhibits a checkerboard-like order (Figure 2b); in addition to the peaks due to the magnetic order at (±π, 0), there appears now an additional pair of peaks at (0, ±π) in the Fourier transformation (Figure 2c). We note that a very similar checkerboard-like AFM order shows up at the surface of samples with much higher levels of excess Fe doping (*x* ≈ 0.2), where the bulk crystal structure is orthorhombic.^6^ Based on the above observations, we deduce that a noticeable amount of strain is already present in the sample.

Further reduction of the sample thickness by two more cleaves results in a sample thickness of roughly 10 μm (see also Supporting Information Note 1 and Figure S1), where the appearance of the surface changes dramatically, see Figure 2d. The surface is now characterized by domains of stripe-like patterns running along one of the two ⟨110⟩ directions separated by domain boundaries running along the crystallographic *a* and *b* directions (Figure 2e). Depending on the stripe orientation, each domain contributes a pair of peaks at (±π, ±π) and (±π, ∓π) to the Fourier transformation (Figure 2f). While one might expect only one of those phases to appear with strain, with the wave-vector parallel to the macroscopic strain direction, it is likely that the strain pattern at the nanometer scale is much more complex due to the formation of monoclinic domains. We find a roughly equal population of the two types of the (π, π)-ordered phases (see Supporting Information Note 2 and Figure S2 for details).

Detailed analysis of an individual domain reveals additional features forming on top of the (π, π)-ordered phase. Shown in Figure 2g, in addition to the image contrast originating from the (π, π) order, there are evenly spaced protrusions present along the stripes, which lead to additional peaks (marked by dashed circles) in the Fourier transformation (Figure 2h). These features arise from two sets of superstructures (see also Figure S3). With their orientations aligned with the (π, π)-ordered phase (Figure S4), one superstructure is characterized by reciprocal lattice vectors  and  and the other by *q*_1,SS2_ = (±2, ∓1) and *q*_2,SS2_ = (±1, ∓2).

To verify the expectation that the strain induces a change in the magnetic order toward the (π, π) order found in pnictides, we have performed spin-polarized STM on a surface region containing both the (π, 0) bicollinear AFM order characteristic of FeTe and the (π, π)-ordered phases. The (π, 0)-ordered phase serves as a calibration reference for the spin-polarization of the tip.^11,18^ To achieve spin sensitivity, we used a ferromagnetic probe tip, prepared by picking up excess Fe atoms directly from the sample surface.^11^ By imaging the same surface location in a magnetic field of 3 T applied in opposite directions along the surface normal, any change in the image contrast can be attributed to surface magnetic order with nonzero components along the crystallographic *c* direction.

**Figure 3:**
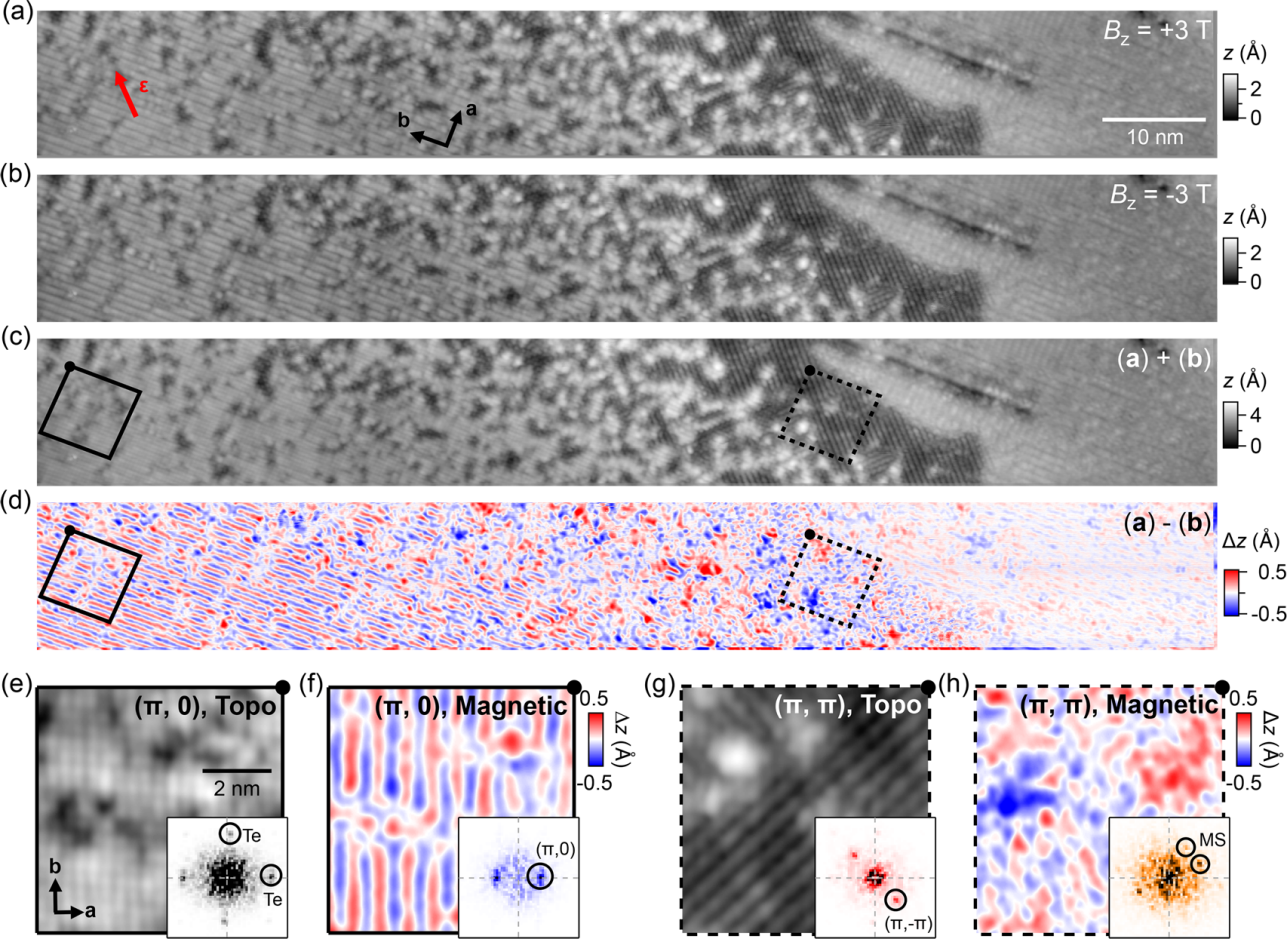
Out-of-plane magnetic structure of the (π, 0)- and (π, π)-ordered phases. (a, b) Topographic images of the surface of a strained Fe_1.1_Te sample recorded with a magnetic tip in applied vertical fields of (a) +3 T and (b) −3 T (*V* = 20 mV, *I* = 0.5 nA). The imaged region comprises both the (π, 0) bicollinear AFM-ordered (left) and (π, π)-ordered phases (right). (c) Addition of images in (a) and (b), showing the purely topographic contrast. (d) Subtraction of image in (a) from that in (b), showing the magnetic contrast. In (c) and (d), solid and dashed squares mark the regions from which the images of the (π, 0)- and (π, π)-ordered phases shown in (e)–(h) are extracted. (e–f) Topographic (e) and magnetic (f) images of the (π, 0) AFM-ordered phase. (g–h) Topographic (g) and magnetic (h) images of the (π, π)-ordered phase. Insets of (e)–(h) show the Fourier transformation of (e)–(h). Peaks associated with the observed orders of different types are highlighted. As circled in the inset of (h), the magnetic superstructure (MS) of the (π, π)-ordered phase has peaks at (±2π/3, ±4π/3) and (±4π/3, ±2π/3).

Figure 3a shows an STM topographic image taken from a surface region comprised of both the (π, 0) bicollinear and (π, π)-ordered phases. The imaged region has a size of (114.3 × 14.3) nm^2^. To achieve the highest possible spatial precision we have collected ultrahigh resolution images in both out-of-plane field directions, with a total pixel number of ∼2 × 10^6^ for each image (see Figure 3, a and b). Following addition of and subtraction between the two images, we have arrived at the corresponding topographic and spin-polarized images, shown in Figure 3, c and d.

Figure 3, e and g, shows close-up topographic images of the (π, 0)- and (π, π)-ordered phases extracted from the large-scale image shown in Figure 3c. The corresponding spin-polarized images are also shown (Figure 3, f and h). The images of the (π, 0)-ordered phase are characteristic of FeTe, showing the square lattice of the surface Te atoms and the bicollinear AFM order along the crystallographic *b* direction.

The topographic image of the (π, π)-ordered phase (Figure 3g) is still dominated by the stripe pattern, whereas the spin-polarized image in Figure 3h shows rather short-ranged magnetic order, thus indicating the absence of long-range magnetic order. The (π, π)-ordered phase hence is predominantly a charge-ordered phase.

The Fourier transformation of the spin-polarized image (Figure 3h) reveals characteristic wave vectors that originate from the short-range magnetic order with wave-vectors of  and , whereas the topographic image is dominated by the (π, π) charge order (inset of Figure 3d).

To find out if the (π, π) order forms as a result of uniaxial strain, we have analyzed the lattice constants between the (π, 0)- and (π, π)-ordered phases, achieved by detecting any change in the ratio of the lattice constants along the crystallographic *a* and *b* directions, *b*/*a*. Analyzing this in reciprocal space, we determine *q*_*a*_/*q*_*b*_ from local Fourier transformations.

**Figure 4:**
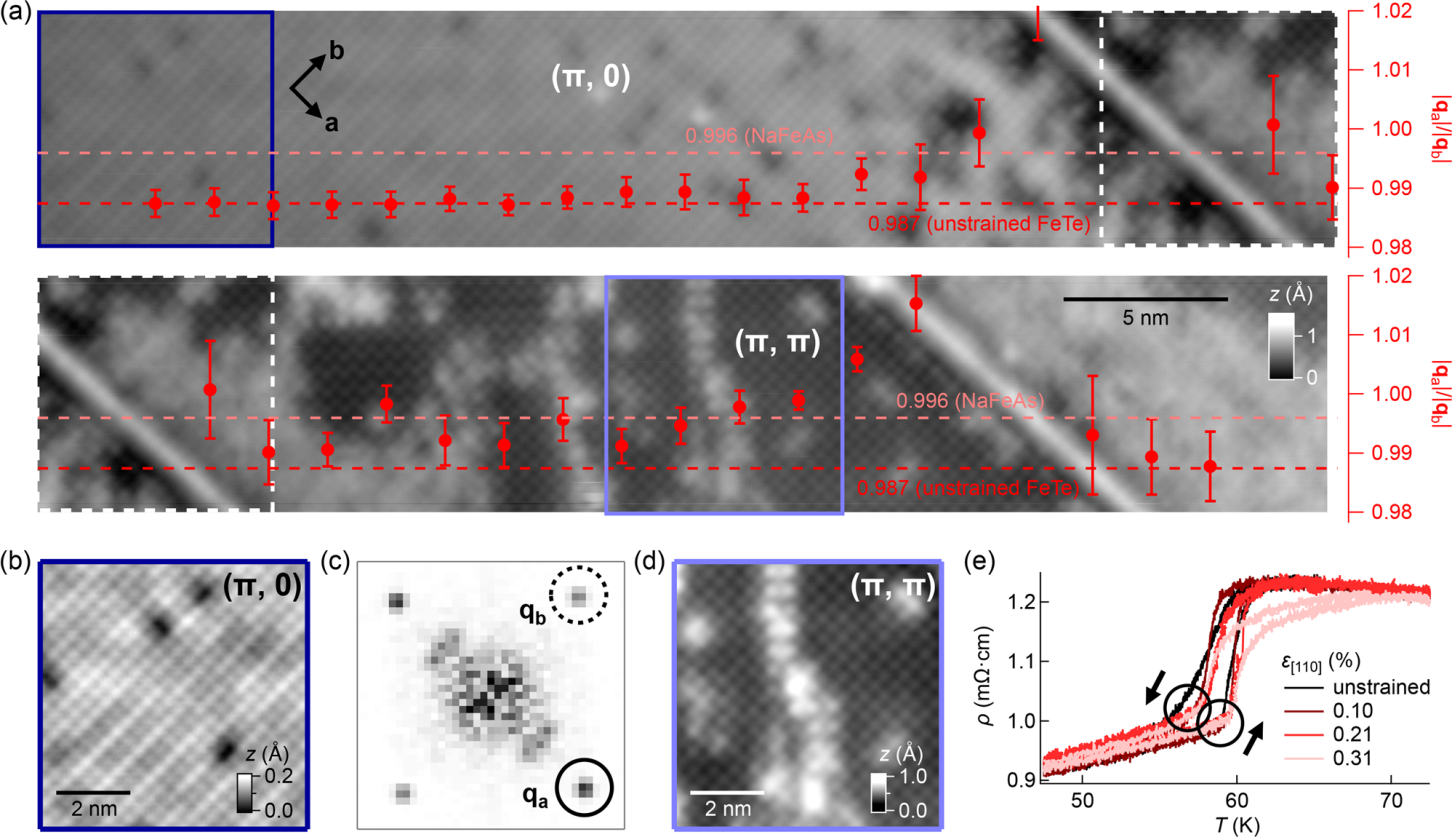
Tensile strain as the origin of the (π, π)-ordered phase. (a) Topographic image taken across a surface region on a strained Fe_1.1_Te sample comprised of both the (π, 0) bicollinear AFM-ordered and (π, π)-ordered phases (*V* = −700 mV, *I* = 500 pA). Due to its high aspect ratio, the image is presented using two separate panels, with the top (bottom) panel showing the left (right) part of the image. Dashed squares indicate the position where the panels overlap. Overlaid scatter plot shows the calculated length ratios, *Q*_*b,a*_ = |**q**_*a*_|/|**q**_*b*_|, at different positions across the image. The *Q*_*b*,*a*_ value at the (π, 0) region is normalized to the value in Fe_1+*x*_Te (0.987), corresponding to that reported by Bao et al.^10^ (b) Zoomed-in image of the (π, 0) order marked by a dark blue square in (a). (c) Fourier transformation of (b). Circles mark the lattice Bragg peaks, **q**_*a*_ and **q**_*b*_. To calculate *Q*_*b*,*a*_, the exact locations of **q**_*a*_ and **q**_*b*_ in momentum space were determined from numerical fitting using a 2D Gaussian function. (d) Zoomed-in image of the (π, π) order marked by a light blue square in (a). (e) Resistivity ρ as a function of temperature measured from a Fe_1.1_Te sample at increasing tensile strain. The hysteresis loop sharpens up at ϵ = 0.1%, then rebroadens again as the strain value increases further.

To accomplish this, we have recorded a high-aspect ratio, high-spatial resolution image from a surface region containing both the (π, 0) and (π, π) phases. Shown in Figure 4a, the recorded image consists of ∼700,000 pixels, covering a surface region of (71.4 × 7.1) nm^2^.

The data points that overlay the topographic image in Figure 4a are the ratios of *q*_*a*_/*q*_*b*_ calculated at different locations across the whole surface region. They were obtained by first measuring the lattice constants |**a**| and |**b**| in a square-shaped moving window in Figure 4a from the corresponding peaks (**q**_**a**_ and **q**_**b**_) in the Fourier transformation (see Figure 4, b and c, for the blue square in Figure 4a). In Figure 4a, our data show that the (π, 0)-ordered region has a *q*_*a*_/*q*_*b*_ ratio very close to that reported by Bao et al. from neutron powder diffraction (0.987),^10^ whereas in the (π, π)-ordered region (Figure 4d), we determine a value of 0.996, very close to the orthorhombicity in Fe-pnictides.^19,20^ We have verified the fidelity of our method to determine the lattice constants through analysis of the lattice constants in two domains on either side of a twin boundary in unstrained Fe_1+*x*_Te (see Figure S5). This analysis demonstrates that the (π, π)-ordered phase is associated with a significant distortion of the surface layer. To verify the impact of the strain on the bulk magnetic order, we have measured the response of the resistivity of uniaxially strained Fe_1.1_Te. Despite higher levels of strain of up to 0.4% achieved on the bulk samples, the resistivity does not show any significant change in the transition to the magnetically-ordered phase (Figure 4e and Figure S6). This suggests that the (π, π) phase that we find here is a reconstruction occurring in the surface layer as a result of the applied strain, or that the phase exists only as a minority phase in the bulk. Previous measurements on the unstrained Fe_1+*x*_Te sample have reported magnetic surface reconstruction, where spins tilt out of the surface and acquire a finite angle with the *b* direction.^21^ We therefore expect that strain can have a different impact on the bulk and surface magnetic structure, and any change in the bulk only follows at higher levels of strain. This raises the exciting possibility that the surface layer responds more sensitively to uniaxial strain than the bulk of the material.

It is worth noting that the (π, π)-ordered phase exhibits striking similarities with some of the Fe pnictides but also some differences. In addition to having a similar ratio of the lattice constants in the *a* and *b* directions (see Figure S7), some of the defects in the (π, π)-ordered phase exhibit the same dumbbell-like appearance as those found on the surface of LiFeAs.^22^ As for the electronic structure, the d*I*/d*V* spectrum obtained from the (π, π)-ordered phase is highly asymmetric with respect to zero bias, which is very different from that of the (π, 0)-ordered phase of the unstrained sample. Such asymmetry in the tunneling spectra was also observed in LiFeAs,^23,24^ although the (π, π)-ordered phase uncovered here is not superconducting (Figure S7).

## Conclusions

Our measurements demonstrate *in situ* strain manipulation of the magnetic order in the nonsuperconducting parent compound of the iron chalcogenide superconductors. Application of uniaxial strain leads to the formation of a new phase in the surface layer that exhibits a markedly distinct appearance from that of unstrained FeTe. The STM images reveal a (π, π) charge order and short-range magnetic order in the strained regions of the surface. They further demonstrate the first step toward a strain-driven control of quantum phases, where the response of the material is not just a linear response expected from the displacement of the atoms but the material is driven into an entirely new phase that does not exist in the unstrained material.

## Experimental Section

### Strain Tuning Device for STM

The strain device used in the STM measurements comprises a brass body and a piezoelectric actuator glued to the brass body with its side-wall facing upward.^16^ Application of a positive (negative) voltage across the leads of the actuator leads to expansion (contraction) along the longitudinal direction of the actuator and contraction (expansion) along the transverse direction. Fe_1.1_Te samples were glued onto the side-wall of the actuator with the Fe–Fe [110] direction aligned parallel to the longitudinal direction of the actuator. Epotek H20E conductive epoxy was used for sample gluing. Clean surfaces were achieved by gluing a rod on top of the sample also using Epotek H20E conductive epoxy, which was knocked off at an *in situ* cleaving stage at ∼20 K.^25^ To maximize strain achieved at the surface of the material, we have studied a number of cleaves of the same sample with a starting thickness of ∼50 μm, as the strain present at the surface depends on the sample thickness. Anisotropic thermal contraction/expansion of the piezoelectric actuator leads to a strain at its interface with the FeTe sample of ∼0.3%, providing an upper boundary for the levels of strain achieved.

### Scanning Tunneling Microscopy and Spectroscopy (STM/S)

STM/S measurements were performed using a home-built, low-temperature STM instrument that operates at a temperature as low as 1.6 K.^25,26^ PtIr tips were used, which were conditioned by field-emission on a gold single crystal. Tunneling spectroscopy measurements were performed using standard lock-in technique, with the frequency of bias modulation set at 413 Hz. Ferromagnetic tips used for SP-STM measurements were prepared by picking up the interstitial Fe atoms from the Fe_1+*x*_Te sample in STM to create a ferromagnetic cluster of Fe atoms at the tip apex.^11,18^ All STM images were taken with a magnetic tip unless stated otherwise.

### Sample Growth

Single crystals of Fe_1+*x*_Te were grown by the self-flux method.^27,28^ The excess iron concentrations *x* reported here have been determined using both energy-dispersive X-ray (EDX) analysis and X-ray diffraction (XRD, see Figure S9). The XRD measurement was performed at the Diamond Light Source using the I12-JEEP high-energy X-ray beamline.^29^ Via the lattice constant, XRD at room temperature provides a very precise estimate of *x*.^5^ Throughout the main text, the excess iron concentration of bulk samples (i.e., before removal of surface excess iron) refers to the off-stoichiometric part *x* of the composition of the material, which in principle can originate either from interstitial iron or a tellurium deficiency.

### Strain Setup for Transport Measurements

A piezoelectric-based stress cell (FC100 from Razorbill Instruments Ltd.) was used for *in situ* control of tensile/compressive strain (see Figure S8a for its photograph). The Fe_1.1_Te single-crystal sample was glued using Stycast 2850FT two-part epoxies onto a grade-V titanium bow-tie-shaped sample platform (thickness = 50 μm, sample mounting area = (75 × 100) μm^2^, see Figure S8b for its schematic), which was then glued onto the clamps of the stress cell also with Stycast 2850FT. With the in-plane crystal orientations of the Fe_1.1_Te sample determined using electron backscatter diffraction, the rectangular bar-shaped crystals were mounted onto the sample platform such that the applied strain on the crystal was along the [110] direction. Before they were mounted onto the sample platform, the Fe_1.1_Te crystals were cleaved using scotch tape to reduce the crystal thickness to below ∼50 μm. The horizontal force applied to the sample platform was determined by measuring the change in capacitance of a distance sensor for a given applied voltage on the piezo-stacks, with the value of strain calculated using the Young’s modulus of 113 GPa for grade-V Ti. Transport measurements were carried out in the standard four-point configuration, and electrical contacts were made using room temperature cured silver epoxy. Strain was applied at a temperature of 65 K, and resistance was measured followed by cooling and warming, sequentially.

## Supporting Information Available

Measurement of sample thickness using high-energy X-ray imaging, population analysis of the (π, π)-ordered phases in different orientations, Fourier component simulation on the (π, π) order and the superstructure, lattice distortion measurement across different monoclinic domains on unstrained Fe_1+*x*_Te, computational details, and supporting figures (PDF)

## Notes

Underpinning data will be made available at: Yim, C. M., Panja, S., Trainer, C. W. J., Topping, C. V., Heil, C., Gibbs, A., Magdysyuk, O., Tsurkan, V., Loidl, A., Rost, A. W., Wahl, P., 2021, Strain-stabilized (π,π) order at the surface of FeTe (dataset). Dataset. University of St Andrews Research Portal. https://doi.org/10.17630/50ccad17-9f3e-4806-a78f-38e56c8cea59
