# Supplementary material for: Strain-Stabilized (π, π) Order at the Surface of Fe1+xTe
Source: Nano Lett. 2021 Apr 2;21(7):2786–92. doi: 10.1021/acs.nanolett.0c04821 (PMC8050823; doi:10.1021/acs.nanolett.0c04821)
Supplement: Supplementary file 1 — nl0c04821_si_001.pdf [file nl0c04821_si_001.pdf]

Supporting Information for 'Strain-stabilized (π, π) order at the surface of $Fe_{1+x}Te$'

Chi Ming Yim,[1,2] Soumendra Nath Panja,[1] Christopher Trainer,[1] Craig Topping,[1] Christoph Heil,[3] Alexandra S. Gibbs,[4,5,6] Oxana V. Magdysyuk,[7] Vladimir Tsurkan,[8,9] Alois Loidl,[8] Andreas W. Rost,[1] and Peter Wahl[1]

[1]*SUPA, School of Physics and Astronomy, University of St Andrews, North Haugh, St Andrews, Fife, KY16 9SS, UK*

[2]*Tsung Dao Lee Institute and School of Physics and Astronomy, Shanghai Jiao Tong University, Shanghai, 200240, China*

[3]*Institute of Theoretical and Computational Physics, Graz University of Technology, NAWI Graz, 8010 Graz, Austria*

[4]*ISIS Neutron and Muon Source, STFC Rutherford Appleton Laboratory, Didcot, OX11 0QX, UK*

[5]*School of Chemistry, University of St Andrews, North Haugh, St Andrews, KY16 9SA, UK*

[6]*Max Planck Institute for Solid State Research, Heisenbergstrasse 1, 70569 Stuttgart, Germany*

[7]*Diamond Light Source Ltd., Harwell Science and Innovation Campus, Didcot, OX11 0DE, UK*

[8]*Center for Electronic Correlations and Magnetism, University of Augsburg, D-86159 Augsburg, Germany*

[9]*Institute of Applied Physics, Academiei 5, MD 2028, Chisinau, Republic of Moldova*

(Dated: March 25, 2021)

This Supporting Information includes:

- Notes 1-5
- Figures S1-S12

SUPPORTING NOTES

1. Measurement of sample thickness using high-energy X-ray imaging

Following the STM measurement of the (π, π) ordered phase, we have employed high energy X-ray imaging at Beamline I12, Diamond Light Source (UK) to measure the sample thickness. Figure S1a shows the cross-section high-energy X-ray image of the piezo-stack on which the (π, π) ordered phase sample was glued. The piezo-stack has a length of 3mm along the horizontal direction in the image, while the $Fe_{1.1}Te$ sample, glued onto the longitudinal face of the piezo-stack, is nearly invisible. To provide an estimate, in Figure S1c we show the first derivative image of Figure S1a, from which we estimated the sample thickness to be $12 \pm 12\mu$m. As reference, in Figure S1b we show the cross-section image of another piezo-stack glued with a fresh $Fe_{1.1}Te$ sample. Clearly visible in the image, this sample has a measured thickness of $50 \pm 12\mu$m (Figure S1d).

2. Population analysis of the (π, π) ordered phases in different orientations

The (π, π) ordered phase formed on the strained $Fe_{1.1}Te$ sample can be present in two different orientations. Here we studied their relative population.

Figure S2a shows a $(113\text{nm})^2$ topographic image taken from the surface of a strained $Fe_{1.1}Te$ sample that exhibits the (π, π) order. In the image, domains of the (π, π) order are separated by line defects running along the crystallographic a and b directions of $Fe_{1.1}Te$, respectively. Shown in the Fourier transformation (Figure S2b), depending on their orientations, each type of the (π, π) ordered phase leads to a pair of peaks, located either at $(\pm\pi, \pm\pi)$ (marked by red circles) or $(\pm\pi, \mp\pi)$ (marked by blue circles) in Fourier space. By applying a band-filter to the image in Figure S2a, we have generated a distribution map for the two types of domains of the (π, π) order, shown in Figure S2c. Inside the distribution

map, regions of red (blue) color represent the domains of the (π,π) order characterized by wave-vectors of $(\pm\pi,\pm\pi)$ $[(\pm\pi,\mp\pi)]$ in Fourier space (Figure S2b).

Analysis of their populations reveals that the (π,π) and $(\pi,-\pi)$ ordered phases populate $\sim 53.7\%$ and $\sim 42.7\%$ of the total area respectively (Figure S2d). The remaining regions (white-colored) are those occupied by line-defects or step-edges.

3. Fourier component simulation on the (π,π) order and the superstructures

To account for the appearance and long range order of the (π,π) ordered phase and the associated superstructures in STM, we have performed numerical simulations which we will describe as follows.

First, we simulated one-by-one each of the Fourier components that altogether form the image contrast of the (π,π) ordered phase of the strained $Fe_{1.1}Te$ samples as shown in Figure 2g. These include the components of the one-Fe unit cell (Figure S3a), Te unit-cell (Figure S3b), (π,π) order (Figure S3c), and those of the superstructures (Figure S3, d and e). All the long range orders reported here are defined relative to the one-Fe unit cell. As such, the Te unit cell has a $c(2\times 2)$ structure, the (π,π) order has a spatial periodicity of $2a_{\mathrm{Fe}}$ along one of the Fe-Fe directions, and the superstructures have unit cells of $c(12\times 4)$ and $c(6\times 2)$ respectively. By adding all the components together with their corresponding weights, we have formed a composite image (Figure S3f), whose contrast resembles that of the (π,π) ordered phase image shown in Figure 2g in the main text. The corresponding Fourier transformation, shown in Figure S3f, is in excellent agreement with the experimental counterpart shown in Figure 2h in the main text.

4. Lattice distortion measurement across different monoclinic domains on unstrained $Fe_{1+x}Te$

We have developed a methodology to detect change(s) in the lattice constants across different regions on the surface of the strained $Fe_{1+x}Te$ sample. Below we demonstrate how this methodology works.

Figure S5a shows a topographic STM image recorded from the surface of an unstrained $Fe_{1.1}Te$ crystal. The imaged region comprises four differently oriented domains of the mon-

oclinic crystal structure of $Fe_{1+x}Te$, as also visualized by the differently colored shadings in Figure S5b. As described by Warmuth *et al.*,[1] the unit cells in the yellow (mid blue) and green (light blue) domains are oriented oppositely along the crystal c axis, while those in the green (yellow) and light blue (mid blue) domains are separated by a right angle. As a consequence, a flip in the length ratio of the unit cell vectors $Q_{2,1}$, defined as $Q_{2,1} = |\mathbf{q_1}|/|\mathbf{q_2}|$ [where $\mathbf{q_1}$ and $\mathbf{q_2}$ are the unit cell vectors of $Fe_{1+x}Te$ in momentum space (marked by blue and red circles in Figure S5c)], is expected when measured across the different monoclinic domains.

The monoclinic unit cell of $Fe_{1+x}Te$ has lattice constants along the crystallographic a and b directions of 3.835 and 3.784 Å, respectively. This leads to a length ratio $Q_{b,a} = b/a$ of 0.987. When measured across any two of the monoclinic domains which are separated by a right angle, a flip in the $Q_{b,a}$ value between 0.987 to 1.013, or vice versa, is expected.

Figure S5d shows a scatter plot of the $Q_{2,1}$ values measured across the four different monoclinic domains shown in Figure S5a. Here, we note that the topographic image in Figure S5a was taken at an angle where the $Fe_{1+x}Te$ unit cell is oriented at 45° with respect to the slow scan direction. Imaging in this way, any systematic error due to the anisotropy between the slow and fast scan directions will impact equally on both lattice constants a and b, and hence gets cancelled out when $Q_{2,1}$ is calculated.

We present our $Q_{2,1}$ data measured from different locations across all four monoclinic domains shown in Figure S5a, from images taken in both the Forward (from left to right) and Backward (from right to left) fast-scan directions, respectively. The data, determined from the Forward and Backward scan images, are presented as red and blue markers respectively in the scatter plot (Figure S5d). Shown in Figure S5d, the two data-sets overlap almost perfectly to each other, indicating that the $Q_{2,1}$ values obtained in this way are consistent and accurate. We note that there still exists a small but non-negligible hysteresis effect along the slow scan direction, manifesting as a constant offset in all measured $Q_{2,1}$ values. To account for this, we have manually shifted all the measured values (including those in black, which correspond to the average values of those from the Forward and Backward images) so that their mean equals one. As a result, all the data-points calculated from the monoclinic domains on the left of the image have $Q_{2,1}$ values very close to the literature value of 0.987,[2] while those on the right have $Q_{2,1}$ values very close to 1.013, with uncertainties of less than 0.5%.

Our example demonstrates the accuracy of our methodology for detecting tiny changes in atomic distances across the surface in systems with different unit cell domains. In the main text, we have employed this method to establish uniaxial strain to be the origin for the (π, π) ordered phase uncovered in the strained $Fe_{1+x}Te$ samples. See Figure 4 in the main text for details.

5. Computational Details

The density functional theory (DFT) calculations reported in this work were performed in the generalized gradient approximation[3] as implemented in the QUANTUM ESPRESSO package.[4] To describe the $(\pi, 0)$ bicollinear and (π, π) magnetic orders of FeTe in the same cell, a 2×2×1 super-cell is considered, with crystal structure details and atomic positions taken from experiment,[5] and then further fully relaxed. After changing the lattice constants to simulate a certain uniaxial strain, the internal atomic positions are again relaxed to ensure that they are in their equilibrium position. We used projector augmented-wave pseudo-potentials[6] with semi-core states included for both Fe and Te. A kinetic energy cutoff of 60 Ry was chosen for the wave functions, an energy cutoff of 720 Ry for the charge density, and we used a Methfessel-Paxton[7] smearing of 0.01 Ry. The electronic Brillouin zone of the magnetic super-cell was sampled using a 6×6×9 Monkhorst-Pack[8] **k**-mesh.

Supporting References

(1) Jonas Warmuth, Martin Bremholm, Philip Hofmann, Jens Wiebe, and Roland Wiesendanger. Domain imaging across the magneto-structural phase transitions in $Fe_{1+y}Te$. *npj Quantum Materials*, 3(1):21, 2018.

(2) Wei Bao, Y. Qiu, Q. Huang, M. A. Green, P. Zajdel, M. R. Fitzsimmons, M. Zhernenkov, S. Chang, Minghu Fang, B. Qian, E. K. Vehstedt, Jinhu Yang, H. M. Pham, L. Spinu, and Z. Q. Mao. Tunable $(\delta\pi, \delta\pi)$-type antiferromagnetic order in α-Fe(Te,Se) superconductors. *Phys. Rev. Lett.*, 102(24):247001, 2009.

(3) John P. Perdew, Kieron Burke, and Matthias Ernzerhof. Generalized gradient approximation made simple. *Phys. Rev. Lett.*, 77(18):3865–3868, 1996.

(4) Paolo Giannozzi, Stefano Baroni, Nicola Bonini, Matteo Calandra, Roberto Car, Carlo Cavazzoni, Davide Ceresoli, Guido L Chiarotti, Matteo Cococcioni, Ismaila Dabo, Andrea Dal Corso, Stefano de Gironcoli, Stefano Fabris, Guido Fratesi, Ralph Gebauer, Uwe Gerstmann, Christos Gougoussis, Anton Kokalj, Michele Lazzeri, Layla Martin-Samos, Nicola Marzari, Francesco Mauri, Riccardo Mazzarello, Stefano Paolini, Alfredo Pasquarello, Lorenzo Paulatto, Carlo Sbraccia, Sandro Scandolo, Gabriele Sclauzero, Ari P Seitsonen, Alexander Smogunov, Paolo Umari, and Renata M Wentzcovitch. QUANTUM ESPRESSO: a modular and open-source software project for quantum simulations of materials. *Journal of Physics: Condensed Matter*, 21(39):395502, sep 2009.

(5) Shiliang Li, Clarina de la Cruz, Q. Huang, Y. Chen, J. W. Lynn, Jiangping Hu, Yi-Lin Huang, Fong-Chi Hsu, Kuo-Wei Yeh, Maw-Kuen Wu, and Pengcheng Dai. First-order magnetic and structural phase transitions in $Fe_{1+y}Se_xTe_{1-x}$. *Phys. Rev. B*, 79:054503, Feb 2009.

(6) G. Kresse and D. Joubert. From ultrasoft pseudopotentials to the projector augmented-wave method. *Phys. Rev. B*, 59:1758–1775, Jan 1999.

(7) M. Methfessel and A. T. Paxton. High-precision sampling for brillouin-zone integration in metals. *Phys. Rev. B*, 40:3616–3621, Aug 1989.

(8) Hendrik J. Monkhorst and James D. Pack. Special points for brillouin-zone integrations. *Phys. Rev. B*, 13:5188–5192, Jun 1976.

(9) Cevriye Koz, Sahana Rößler, Alexander A. Tsirlin, Steffen Wirth, and Ulrich Schwarz. Low-temperature phase diagram of $Fe_{1+y}Te$ studied using x-ray diffraction. *Phys. Rev. B*, 88(9):094509, September 2013.

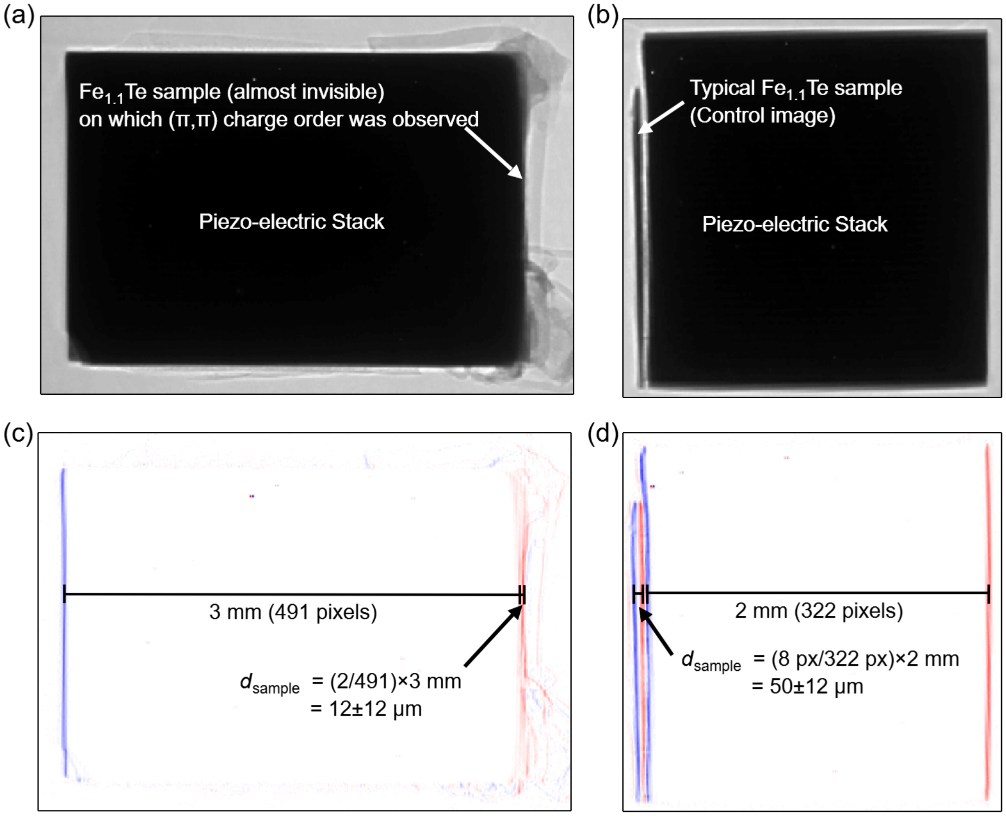

Figure S1. (a) Cross-section hard X-ray image taken from the piezo-stack glued with the $Fe_{1.1}Te$ sample on which the (π, π) charge-ordered phase was observed. (b) As (a), taken from the piezo-stack glued with a fresh $Fe_{1.1}Te$ sample with moderate sample thickness. (c)-(d) Differentiated images of (a)-(b), from which the sample thicknesses were determined.

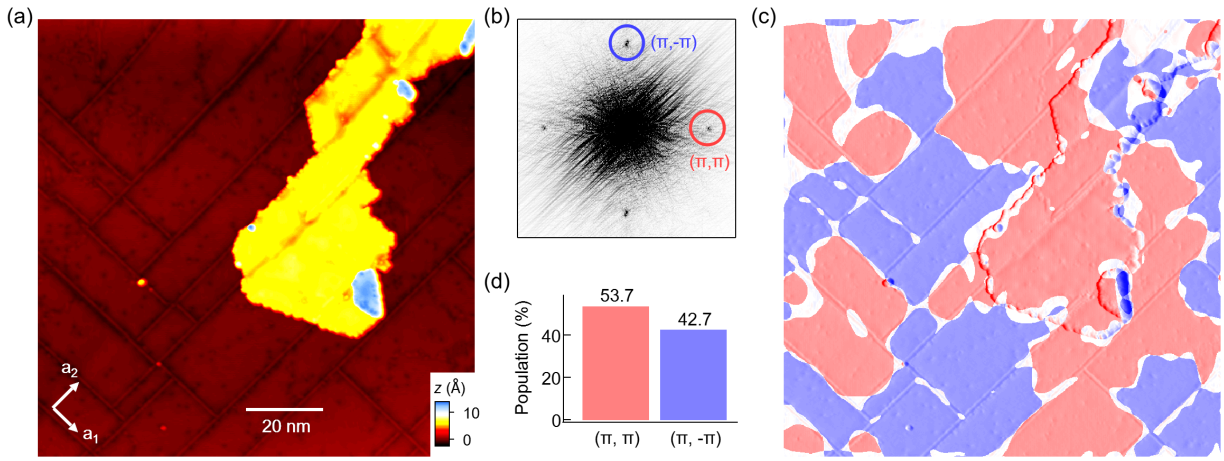

Figure S2. Relative population of the (π,π) phases at different orientations. (a) Topographic STM image of the (π,π) ordered phase formed on the surface of a strained $Fe_{1+x}Te$ sample. The scanned region comprises domains of the (π,π) ordered phases in two different orientations, separated by domain boundaries ($V = 0.2$V, $I = 0.1$nA) (b) Fourier transformation of (a). Red and blue circles mark the peaks associated with the domains of (π,π) order characterized by wave-vectors of (π,π) and $(\pi,-\pi)$, respectively. (c) Distribution map of the (π,π) (red) and $(\pi,-\pi)$ (blue) ordered domains shown in (a). (d) Bar chart showing their relative population.

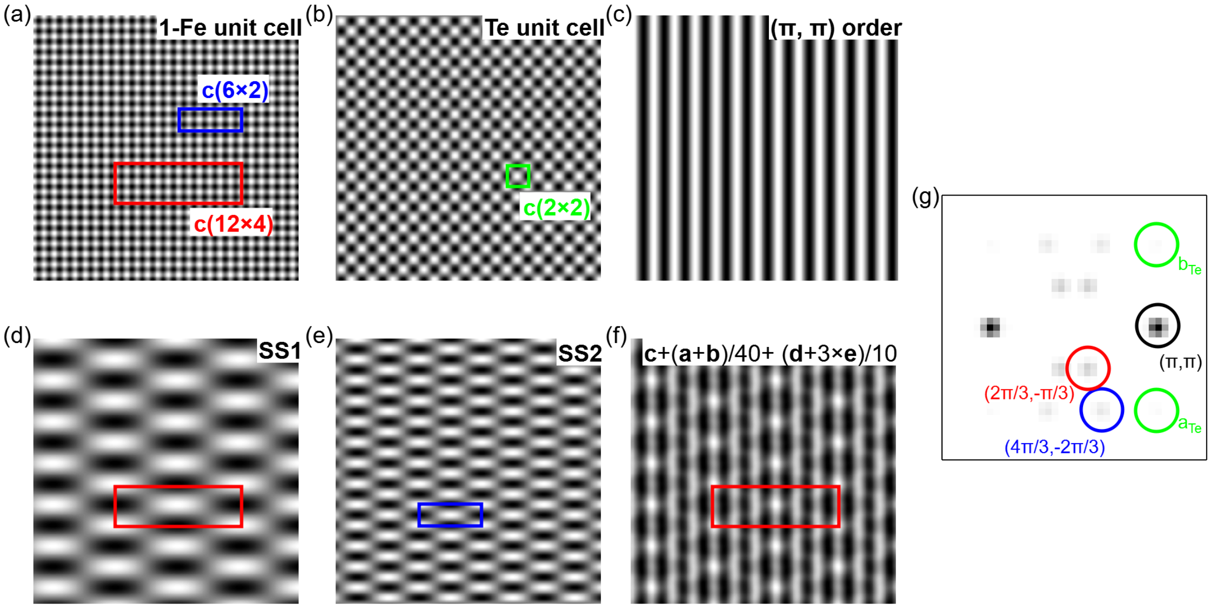

Figure S3. Decomposition of Fourier components of the (π, π) order and the associated superstructure. (a) Lattice grid of one-Fe unit cell. (b) Lattice grid of Te, which has a unit cell of $c(2 \times 2)$ with respect to (a). (c) Simulated image of the (π, π) order, which has a periodicity of $2a$ along the horizontal direction. (d)-(e) Simulated images of superstructures 1 and 2, which have a unit cell of $c(12 \times 4)$ and $c(6 \times 2)$ with respect to (a), respectively (see red and blue rectangles in (a), (d) to (f). (f) Weighted-sum image of those in (a) to (e). (g) Fourier transformation of (f). Peaks associated with the Te unit cell, (π, π) order, and those with the superstructure 1 and 2, are highlighted with green, black, red and blue circles respectively. Note that for the system that exhibits the (π, π) order, the two sets of superstructures forming on top are, relative to the reciprocal unit cell vectors of the Te lattice in the topmost layer, characterized by reciprocal lattice vectors of $q_{1,\mathrm{SS1}} = (\pm 4\pi/3, \mp 2\pi/3)$, $q_{2,\mathrm{SS1}} = (\pm 2\pi/3, \mp 4\pi/3)$ and $q_{1,\mathrm{SS2}} = (\pm 2\pi/3, \mp \pi/3)$, $q_{2,\mathrm{SS2}} = (\pm \pi/3, \mp 2\pi/3)$ respectively.

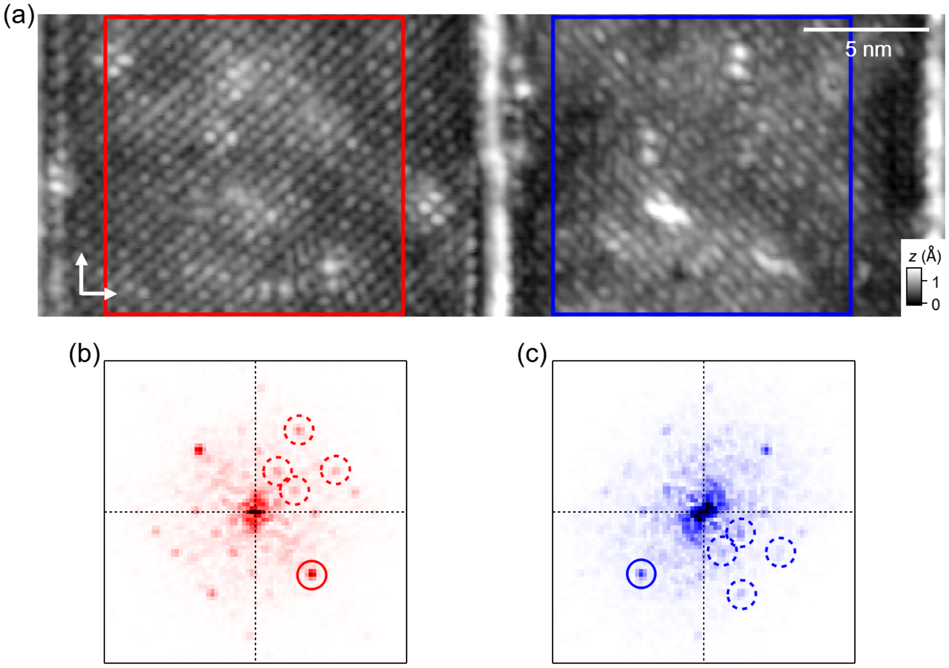

Figure S4. (a) Atomically resolved STM topographic image taken from a surface region comprised of the (π, π) ordered phases of different orientations separated by a domain boundary ($V = 10$mV, $I = 0.1$nA). (b)-(c) Fourier transformation of the imaged regions marked with the red and blue square boxes in (a) respectively. In (b)-(c), solid circles mark the FT peaks associated with the $(\pi, \pm\pi)$ order, dashed circles mark those of the superstructure formed on top of the (π, π) ordered phase.

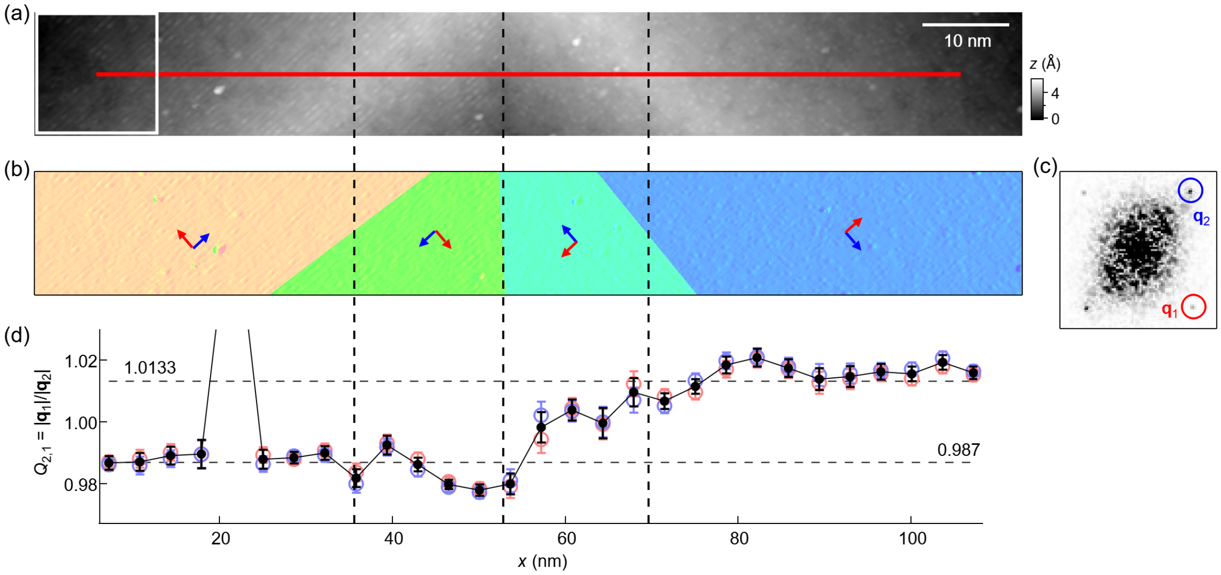

Figure S5. Demonstration of monoclinic distortion in unstrained $Fe_{1.1}Te$. (a) STM image recorded from a surface region of an unstrained $Fe_{1.1}Te$ sample ($V = 25$mV, $I = 0.1$nA). (b) Color map of the four different monoclinic domains in (a). In each of the domains, red (blue) arrows represent crystallographic a (b) direction of FeTe. (c) Fourier transformation of the region marked by a square box in (a). Red and blue circles mark the peaks of the unit cell vectors $\mathbf{q_1}$ and $\mathbf{q_2}$ in Fourier space. (d) Scatter plot of the length ratio $Q_{2,1} = |\mathbf{q_1}|/|\mathbf{q_2}|$ of the reciprocal unit cell vectors $\mathbf{q_1}$ and $\mathbf{q_2}$ measured across the whole image in (a). Red (blue) data points were obtained from the image recorded in the Forward (Backward) scan direction. Black data-points are the average of the two. To correct for the non-negligible hysteresis effect along the slow scan direction, all data-points are vertically offset to an average value of unity. Horizontal dashed lines indicate a length ratio of $Q_{2,1} = 0.987$ and its inverse value of 1.013 respectively, calculated using the values reported by Bao *et al.*.[2]

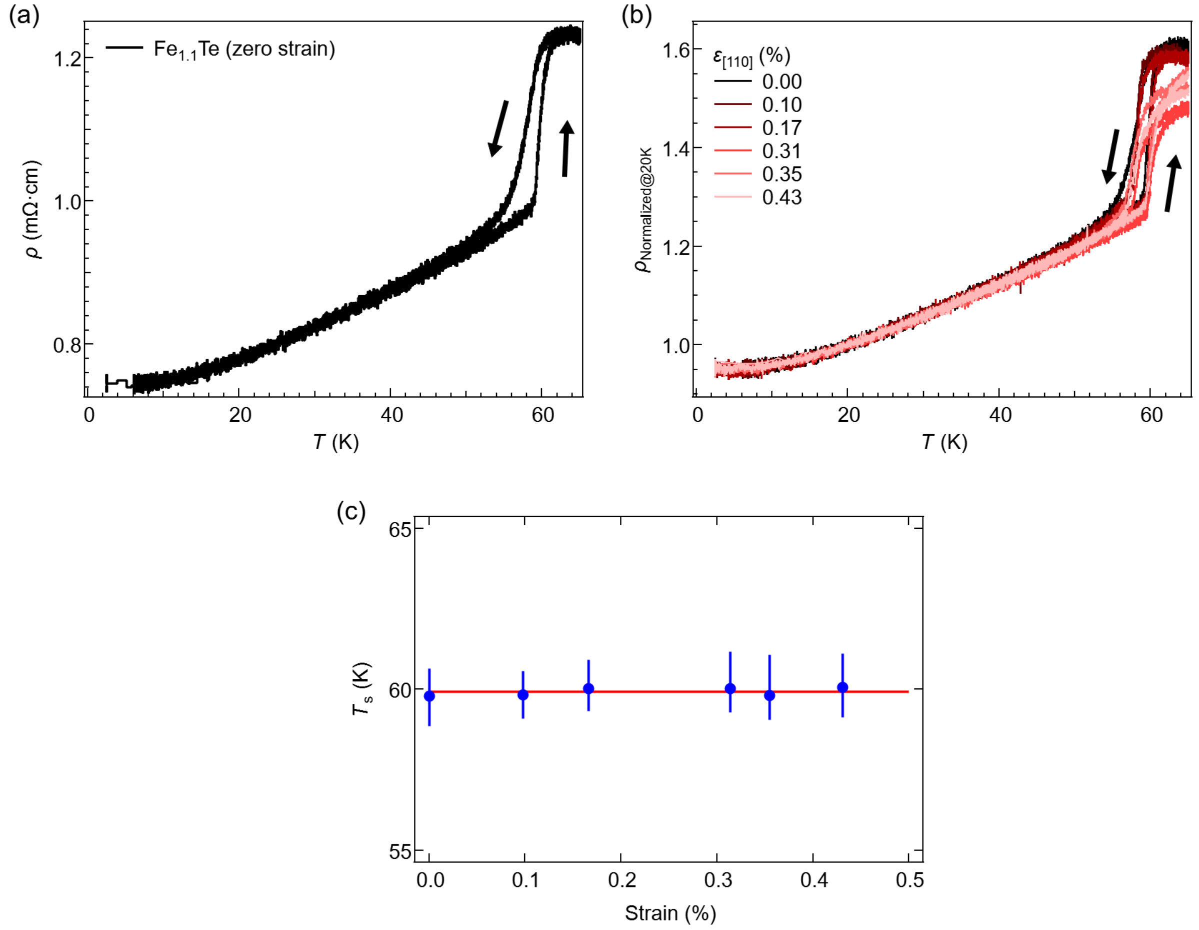

Figure S6. (a) Resistivity (ρ) versus temperature (T) measured from an unstrained $Fe_{1.1}Te$ sample. (b) Normalised $\rho(T)$ curves recorded from a number of $Fe_{1.1}Te$ samples at different applied strain values. All curves are normalized to their resistivity ρ at 20K. (c) Evolution of the structural transition temperature (T_s) of $Fe_{1.1}Te$ with applied strain along the [110] direction. T_s at different strain values were evaluated from the peak positions in the first derivative of the $\rho(T)$ curves ($\mathrm{d}\rho/\mathrm{d}T$) taken in the warm up cycle. Error bars represent the temperature width of the transition determined from the full-width at half maxima (FWHM) of the $\mathrm{d}\rho/\mathrm{d}T$ plot.

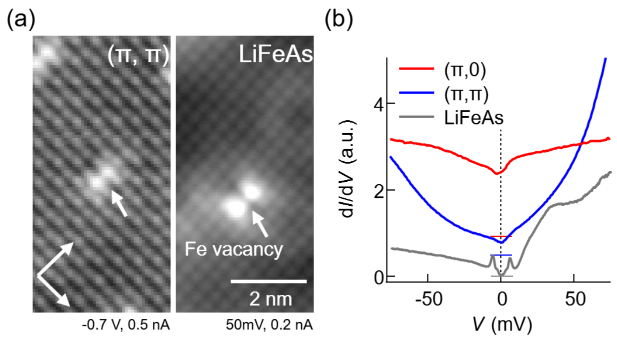

Figure S7. Comparison of the (π, π) ordered phase with the surface of unstrained LiFeAs. (a) STM images taken from (left) the (π, π) ordered phase on strained $Fe_{1.1}Te$ and (right) the surface of LiFeAs, respectively. Arrows indicate the resemblance in appearance between the dumbbell shaped defects present in the (π, π) ordered phase and the Fe-site defects in LiFeAs. (b) $\mathrm{d}I/\mathrm{d}V - V$ spectra taken from the defect-free positions in the (red) $(\pi, 0)$ and (blue) (π, π) ordered phases on the strained $Fe_{1.1}Te$ surface, respectively. The gray spectrum was taken from unstrained LiFeAs. Spectroscopy set-point (V_s, I_s): $70\mathrm{mV}, 0.5\mathrm{nA}$ (strained FeTe), $75\mathrm{mV}, 0.8\mathrm{nA}$ (unstrained LiFeAs). Amplitude of bias modulation V_{mod}: 1mV (strained FeTe), 0.5mV (unstrained LiFeAs).

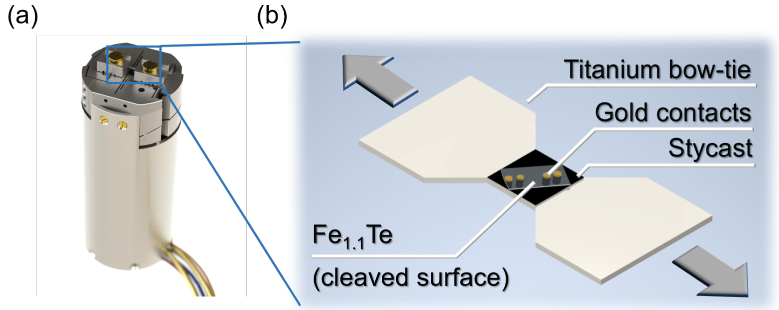

Figure S8. (a) Photograph of the strain-rig employed in the strain-transport measurement (image courtesy of *Razorbill Instruments Ltd*). A rectangle box marks the location of the bow-tie shaped sample platform within the strain-rig. (b) Schematic of the bow-tie sample platform (platform thickness: 50μm).

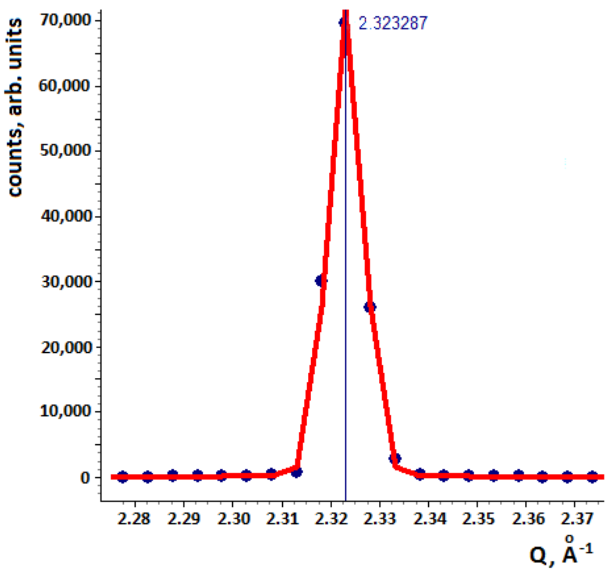

Figure S9. Line-profile taken across the (110) diffraction spot in the x-ray diffraction (XRD) data collected from the $Fe_{1+x}Te$ sample with a sample thickness of $\approx 50\mu$m. The XRD data were taken at a photon energy of 55keV. The measured sample is from the same batch as that where the (π, π) ordered phase was observed. Markers are experimental data, and the red line is numerical fit to the data using Topas, revealing a Q-value of 2.323Å^{-1}. From the Q value, the lattice constant of $Fe_{1+x}Te$ along the crystallographic a direction is determined to be 3.825Å^{-1}, from which the excess Fe concentration (x) within the sample is deduced to be ~ 0.1 according to Koz *et al.*[9]. The XRD data were collected at room temperature.

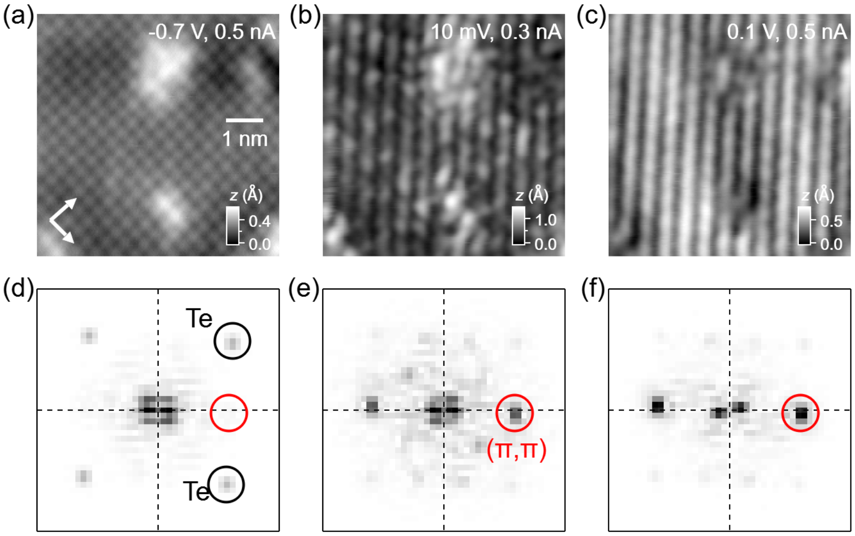

Figure S10. (a)-(c) Atomically-resolved images of the (π, π) ordered phase taken at different bias voltages. (d)-(f) Corresponding Fourier transformations of (a)-(c). In (d), black circles mark the Bragg peaks of the square lattice of Te atoms (with 2 Fe per unit cell). In (d)-(f), red circles mark the (π, π) FT peaks.

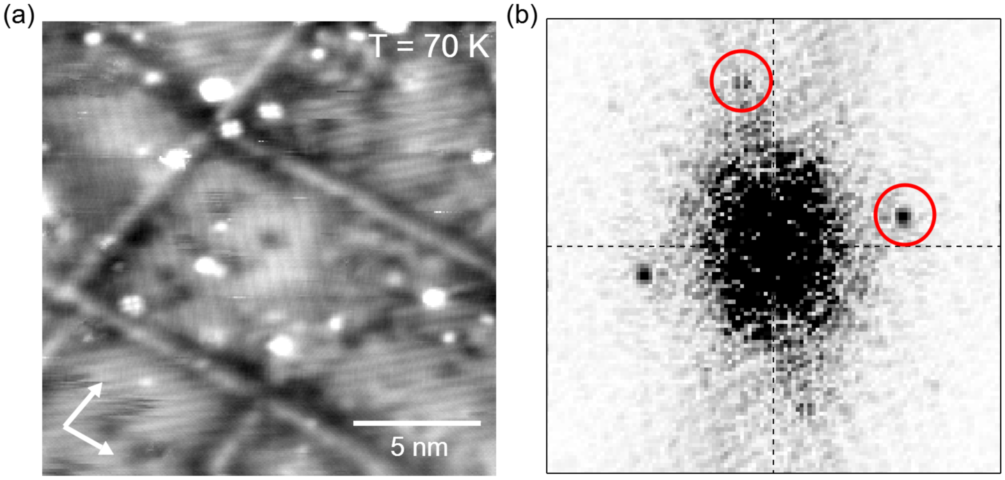

Figure S11. (a) Topographic image of the (π, π) ordered phase taken at $T = 70$K ($V = 0.3$V,$I = 0.5$nA). (b) Fourier transformation of (a). Red circles mark the (π, π) peaks.

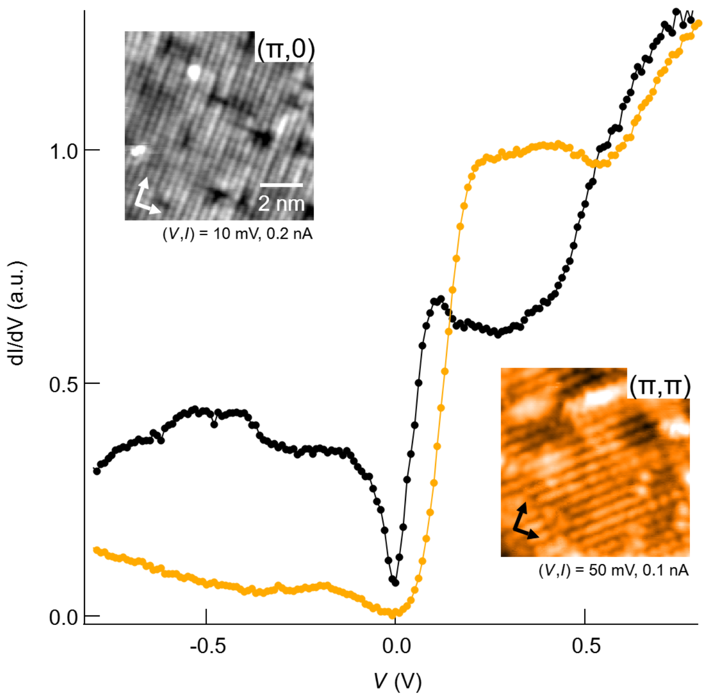

Figure S12. $\mathrm{d}I/\mathrm{d}V$ versus V spectra taken from the defect-free positions of the $(\pi, 0)$ (black) and (π, π) ordered phases (orange) respectively. Spectroscopy set-point (V_s, I_s) : $0.8\mathrm{V}, 0.5\mathrm{nA}$. Amplitude of the bias modulation used: 20mV. Insets: topographic images of the two ordered phases.
